# Five decades of total ankle replacement: from early failures to fourth-generation innovations and future priorities

**DOI:** 10.1007/s00590-025-04634-5

**Published:** 2025-12-29

**Authors:** Gabriele Colò, Federico Fusini, Antonio Mazzotti, Cesare Faldini, Massimiliano Leigheb, Michele Francesco Surace

**Affiliations:** 1 Sant’Anna Hospital, Como, Italy; 2 Multidisciplinary Research Center for Pathology and Surgery of the Musculoskeletal System, Department of Biotechnology and Life Sciences (DBSV), Insubria University, Varese, Italy; 3 Department of Orthopaedic and Traumatology, Orthopaedic and Trauma Centre, University of Turin, Via Zuretti 29, 10124, Turin, Italy; 4 Department of Biomedical and Neuromotor Sciences (DIBINEM), University of Bologna, 40127, Bologna, Italy; 5 1st Orthopaedics and Traumatologic Clinic, IRCCS Istituto Ortopedico Rizzoli, 40136, Bologna, Italy; 6 Orthopaedics and Traumatology Unit, San Gaudenzio Clinic (Monza Polyclinic Group), Via Enrico Bottini 3, 28100, Novara, Italy

**Keywords:** Osteoarthritis, Total ankle replacement, Arthrodesis, Total ankle arthroplasty, Biomechanics

## Abstract

**Background:**

Total ankle replacement (TAR) has evolved considerably over the past five decades as an alternative to arthrodesis for the management of end-stage ankle osteoarthritis. While early TAR designs suffered from high failure rates and limited functional outcomes, subsequent generations have progressively improved implant survivorship and biomechanics. However, determining the optimal prosthesis design for maximizing long-term outcomes remains a challenge, requiring synthesis of analytical performance data and practical guidance for implant selection.

**Methods:**

A targeted analytical review of the literature was conducted by analyzing peer-reviewed publications indexed in PubMed, Scopus, and Web of Science, covering the period from 1970 through July 2025. The search strategy focused on historical development and specifically on design characteristics (fixation, constraint) and their corresponding quantitative outcomes (survivorship, failure rates). Both original studies and systematic reviews were included to assess implant design evolution, materials, fixation techniques, survivorship, and complications. Emphasis was placed on representative implant models from each generation, including dedicated revision systems.

**Results:**

First-generation TARs, such as ICLH (Imperial College London Hospital) and Lord models, demonstrated high failure rates, with only 21% of cases rated as clinically satisfactory at 5.5 years due to constrained designs and cement-related issues. Second-generation systems introduced semi-constrained, mobile-bearing implants with cementless fixation, achieving up to 92% survivorship at 12 years. Third-generation designs, including HINTEGRA and BOX, emphasized anatomical congruence, ligament balancing, and soft tissue preservation, with survivorship ranging from 66 to 92% depending on implant model and follow-up. Fourth-generation implants (e.g., INFINITY, CADENCE, QUANTUM and the lateral-approach Trabecular Metal Ankle System) featured minimal bone resection and improved primary stability, reporting 1–2 year survivorship between 92 and 98%; however, complications such as heterotopic ossification (up to 69%) and talar loosening remain concerns. The development of dedicated revision TAR systems like INVISION represents a recent innovation, though long-term data are limited.

**Conclusions:**

The historical analysis demonstrates a clear performance shift tied to specific design attributes. Specifically, fourth-generation fixed-bearing, cementless designs (reporting survival rates up to 98%) have effectively addressed the mechanical failure and cement-related issues common in earlier constrained and mobile-bearing systems. While challenges persist (such as heterotopic ossification and talar loosening), the data analytically support specific implant choices. Therefore, based on documented survivorship and failure profiles, fourth-generation fixed-bearing, cementless systems are recommended as the primary choice in appropriately selected patients.

**Graphical abstract:**

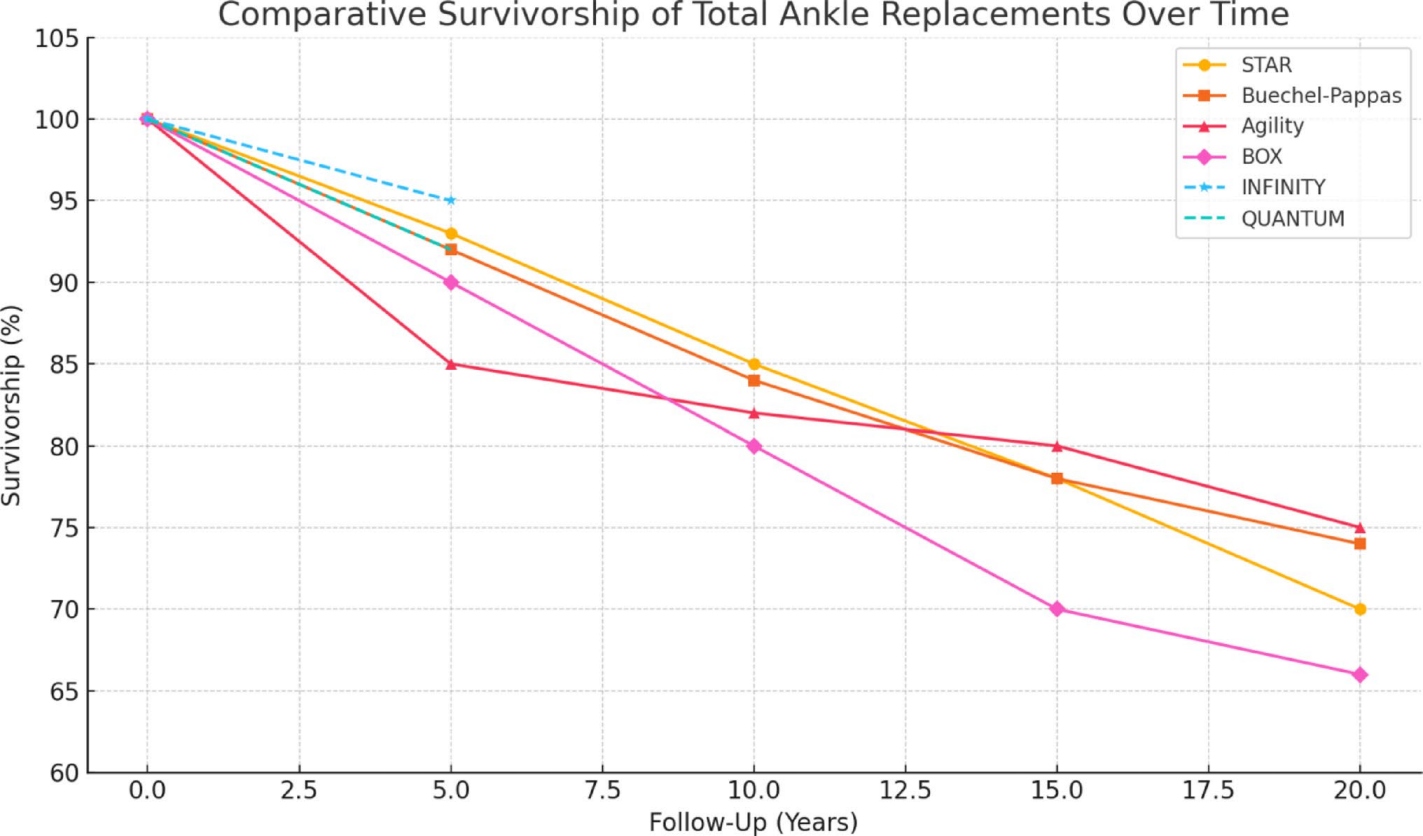

## Introduction

**Fig. 1:**
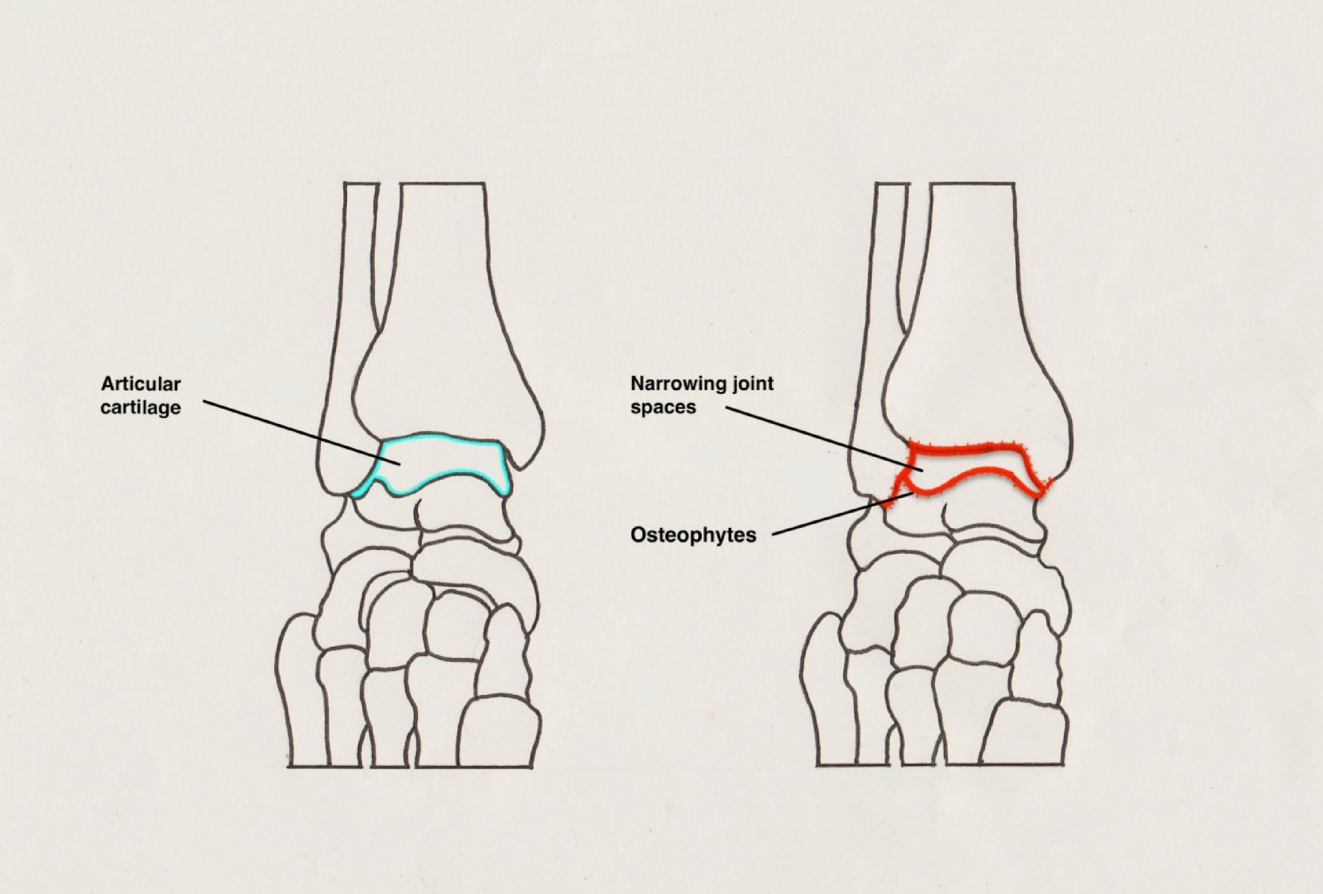
Healthy ankle joint (left) and advanced osteoarthritic joint (right), showing osteophyte formation and joint space narrowing

Ankle osteoarthritis (OA), which affects approximately 1% of the global adult population, predominantly develops as a consequence of previous trauma, accounting for nearly 78% of cases (Fig. 1) [1]. Historically, ankle arthrodesis has been regarded as the gold-standard intervention for end-stage disease, offering reliable pain relief at the expense of joint mobility and with a well-documented risk of adjacent joint degeneration [2, 3]. Motivated by the clinical success of total hip and knee arthroplasty, total ankle replacement (TAR) emerged as a potential motion-preserving alternative. However, the initial iterations of TAR were associated with suboptimal outcomes, largely attributable to rudimentary implant designs, inferior biomaterials, and limited procedural expertise [4, 5].

Substantial advancements in prosthetic design, material science, and operative techniques—particularly during the 1980s and 1990s—have markedly improved the clinical efficacy of TAR. These innovations have led to excellent or good outcomes in over 80% of patients undergoing TAR, compared to approximately 72% of those treated with ankle arthrodesis [6, 7].

The contemporary resurgence of interest in TAR is largely attributed to ongoing improvements in implant architecture, material biocompatibility [8], and enhanced precision in surgical techniques, notably with regard to coronal and sagittal alignment control [9]. Additionally, the long-term morbidity associated with ankle arthrodesis has become increasingly evident. Despite fusion rates ranging from 90 to 93% [10], arthrodesis is frequently complicated by progressive OA in adjacent joints, particularly the subtalar and midtarsal articulations, as highlighted in long-term follow-up studies [11].

Although short- to mid-term outcomes following TAR are generally favorable [12], persistent concerns remain within the literature regarding complications such as delayed wound healing, periprosthetic infections [13], anterior impingement, and the technical challenges associated with salvage or revision procedures [14]. Moreover, clinical success is often closely tied to the surgeon’s experience and procedural volume [15].

This narrative review aims to critically synthesize the analytical performance data derived from the historical progression of TAR. By correlating specific design attributes (e.g., fixed vs. mobile-bearing, cementless vs. cemented) with documented survivorship and failure mechanisms, this analysis seeks to provide objective, evidence-based recommendations for selecting the optimal contemporary prosthesis design.

## Search strategy

A targeted analytical narrative review was conducted to examine the historical development, biomechanical foundations, and to critically analyze the quantitative performance and clinical outcomes of TAR. The literature search was performed across three major databases: PubMed, Scopus, and Web of Science. The time frame encompassed publications from 1970 through July 2025, using a combination of controlled vocabulary and free-text keywords, including “total ankle replacement”, “ankle arthroplasty”, “implant design”, “survivorship”, “complication rates”, and keywords comparing design features (e.g., “fixed vs mobile-bearing”, “cemented vs cementless”) and “revision TAR”. Both original studies and systematic reviews were included. Selection criteria encompassed articles reporting on implant generations, fixation methods, material composition, surgical techniques, and complication profiles. Emphasis was placed on landmark studies representative of each generation of TAR, including those focusing on dedicated revision systems. Studies not published in English or lacking peer-review were excluded. The gathered data were synthesized thematically and chronologically to capture technological evolution and to contextualize clinical outcomes across decades of TAR development. Due to the narrative nature of this review, formal meta-analytical techniques were not applied; however, emphasis was placed on synthesizing quantitative data (survivorship and complication rates) to objectively compare outcomes across implant generations.

## Biomechanics and kinematics

### Anatomical and biomechanical characteristics

The ankle joint is a highly specialized anatomical structure subjected to substantial mechanical loading during locomotion, despite having a considerably smaller articular surface compared to the hip or knee joints [16–18]. This limited contact area results in elevated stress across the joint [19, 20]. Within this context, the talus experiences complex and heterogeneous stress distributions, yet its morphology is biomechanically optimized to accommodate such forces [21, 22]. Notably, the talus possesses greater structural strength than the distal tibia, of which only the most inferior segment comprises dense and robust cortical bone [23–25]. The superior surface of the talus is convex and exhibits an oblique valgus orientation that projects anteriorly and laterally, while the corresponding articular surface of the tibia is anatomically congruent, thereby ensuring optimal joint conformity [26].

### Kinematics of the ankle joint

Due to this specific oblique orientation, dorsiflexion of the ankle is biomechanically coupled with physiological eversion of the foot [27]. Furthermore, ankle joint movement encompasses not only dorsiflexion and plantarflexion but also complex transverse plane motions—namely pronation and supination—facilitated by the lateral rocking of the talus between the medial and lateral malleoli [28, 29]. These mediolateral motions are essential for the distribution and modulation of joint forces across the articular surfaces [30]. Importantly, even minimal displacement of the talus significantly impacts joint congruence: a 1 mm lateral shift results in an approximate 40% reduction in the tibiotalar contact surface area [31].

### Implications for total ankle replacement

Design principles for TAR must accommodate these intricate biomechanical parameters to faithfully replicate physiological joint kinematics. To achieve durable outcomes, TAR systems should promote appropriate ligamentous balance, preserve osseous integrity, and ensure biomechanical compatibility with native joint motion [32]. Considering the marked reduction in contact area caused by minimal talar displacement, implant design must also emphasize precise component alignment to maintain joint congruence and ensure even load distribution. Furthermore, prosthetic geometry should be tailored to reproduce not only sagittal plane motion, but also the coupled mediolateral and transverse plane movements that are critical for physiological ankle function [33].

## History

### First-generation implants

The first attempt at TAR was reported in 1970 by Lord and Marotte (Marseille, France), who utilized a femoral prosthesis stem inserted into the tibia in conjunction with a cemented acetabular cup fixed into the calcaneus, necessitating complete resection of the talus [34]. This pioneering but rudimentary technique yielded modest results, with only 7 out of 25 cases deemed clinically acceptable. Nevertheless, it initiated the conceptualization of various two-component prosthetic models, generally composed of a metal component articulating with a polyethylene element. In most configurations, the polyethylene component was positioned in the tibia and the metallic component in the talus, both secured with cement [35].

Both constrained and unconstrained designs were explored in this early phase [36], each employing differing surface geometries—including spherical, spheroidal, cylindrical, concave-convex, convex-convex, trochlear, and bispherical articulations [37]—to facilitate dorsiflexion, plantarflexion, and inversion-eversion motion. Constrained implants offered enhanced intrinsic stability but limited range of motion (ROM), whereas unconstrained designs allowed greater ROM at the expense of increased stress on surrounding ligamentous structures [38].

A notable example among constrained models is the ICLH (Imperial College London Hospital; London, UK) prosthesis, designed by Freeman in 1972 [39]. This system featured a concave polyethylene tibial surface articulating with a convex metallic talar dome, resected to accommodate a single-axis rotational center. However, excessive constraint often resulted in loosening of the tibial component, while dome resection predisposed the talar component to collapse.

In a clinical study by Bolton-Maggs et al., 62 ICLH prostheses were implanted between 1972 and 1981 [40]. Only 13 cases were deemed satisfactory at a mean follow-up of 5.5 years; however, all 13 were ultimately explanted and converted to arthrodesis.

**Fig. 2:**
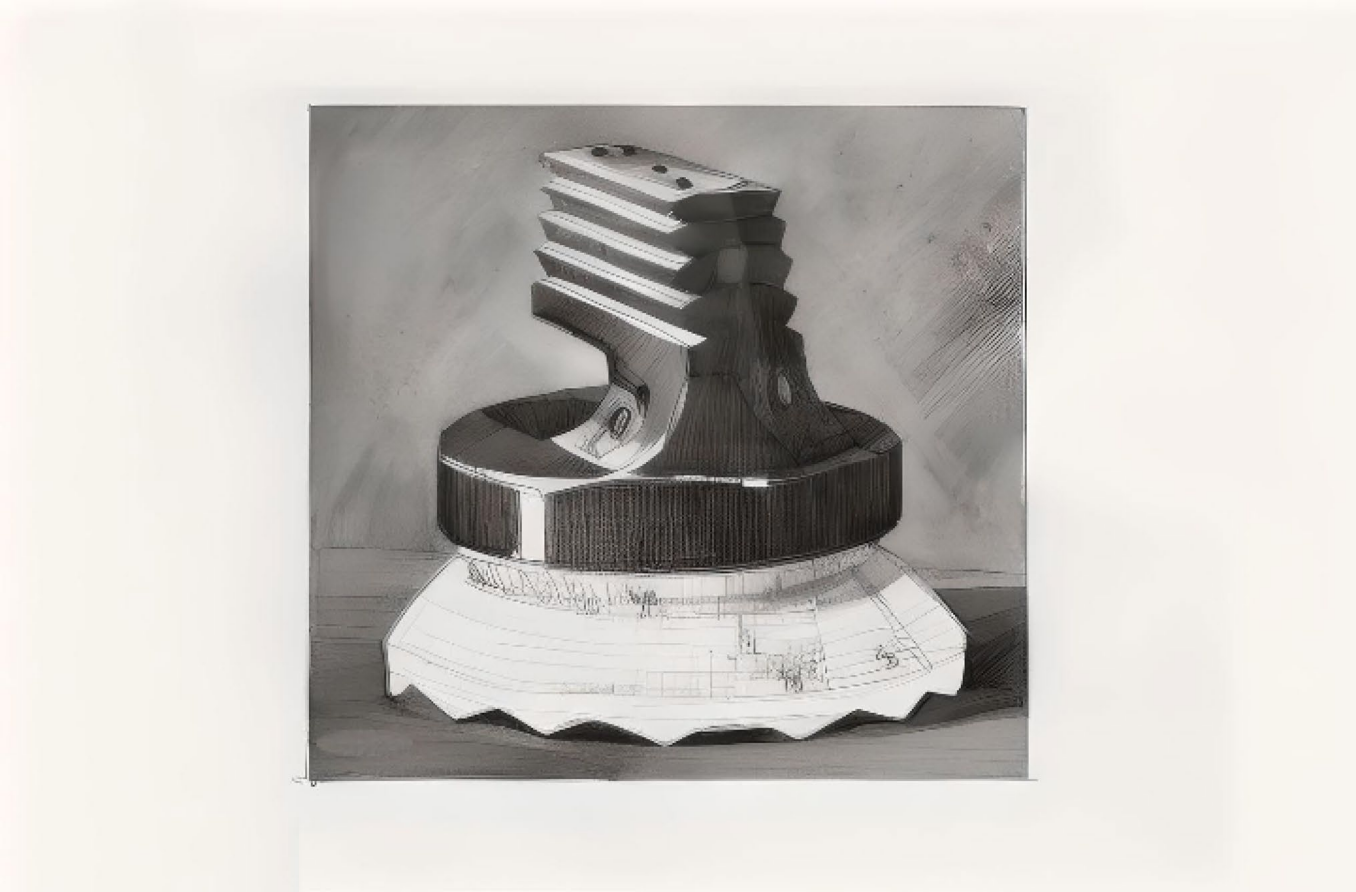
Richard Smith prosthesis: a first-generation, unconstrained ball-and-socket design

Among early unconstrained designs, the Richard Smith prosthesis (Richard Smith design; USA) —introduced in 1975—embodied a spherocentric “ball-and-socket” configuration [41] (Fig. 2). It incorporated a polyethylene talar component with a spherical cap and a metallic tibial counterpart, designed for congruent articulation. While this configuration provided substantial freedom of motion, it also imposed abnormal loads on the ligamentous structures and malleoli, frequently resulting in mechanical failure and collapse of the talar component.

Another critical factor in the high failure rates of first-generation TARs was the reliance on cement fixation, which was prevalent in the 1970s. Cementation required extensive bone resection, thereby weakening structural support and contributing to early loosening and instability [38].

During this era, approximately 15 different two-component fixed-bearing prostheses were developed. Representative constrained systems include the Conaxial (Beck-Steffee; Cleveland Clinic Foundation, Cleveland, OH, USA) [42], Mayo (Mayo Clinic, Rochester, MN, USA) [43], and Oregon (University of Oregon Health Sciences Center, Portland, OR, USA) [44] ankle prostheses. Semiconstrained models included the St. Georg–Buchholz (St. Georg Hospital, Hamburg, Germany) [45], Thompson-Richard (Royal National Orthopaedic Hospital, Stanmore, UK) [46], and Pipino-Calderale (Galeazzi Orthopaedic Institute, Milan, Italy) [47] implants. Among unconstrained variants, key examples include the Lord [48], Smith [41], Newton (University of California, San Francisco, CA, USA) [49], Irvine (University of California, Irvine, CA, USA) [50], and Bath–Wessex (Wessex Orthopaedic Centre, Bath, UK) [51] prostheses.

### Second-generation implants

In the constrained first-generation TAR designs, poor clinical outcomes were largely attributed to the inability of the implants to accommodate the natural variability in ankle joint axis and to dissipate torsional stresses. Conversely, unconstrained models did not yield improved results due to excessive mechanical loading on the surrounding soft tissues, which stemmed from the absence of intrinsic constraint [38]. Most second-generation implants addressed these issues by adopting semi-constrained, cementless configurations.

Virtually all second-generation TAR systems introduced during this era eliminated bone cement use, favouring porous-coated components with hydroxyapatite coatings to facilitate osseointegration and achieve a stable bone–implant interface. These designs also required minimal bone resection. Based on earlier design failures, second-generation prostheses more faithfully replicated native ankle anatomy and kinematics [52]. They can be broadly categorized into two types: two-component fixed-bearing (FB) and three-component mobile-bearing (MB) systems. FB designs include a single articulation between the tibial and talar components, with the polyethylene insert fixed to the tibial baseplate. MB prostheses incorporate a freely mobile polyethylene meniscus that articulates with both tibial and talar components, effectively creating two articular surfaces. The mobile insert reduces shear forces and enhances congruity, thereby minimizing polyethylene wear [53].

Notable prostheses introduced during this period include the STAR (Scandinavian Total Ankle Replacement; Kofoed design, later manufactured by Waldemar Link, Hamburg, Germany) [54], Buechel-Pappas (Endotec, South Orange, NJ, USA) [55], and Agility (DePuy, Warsaw, IN, USA) [56].

**Fig. 3:**
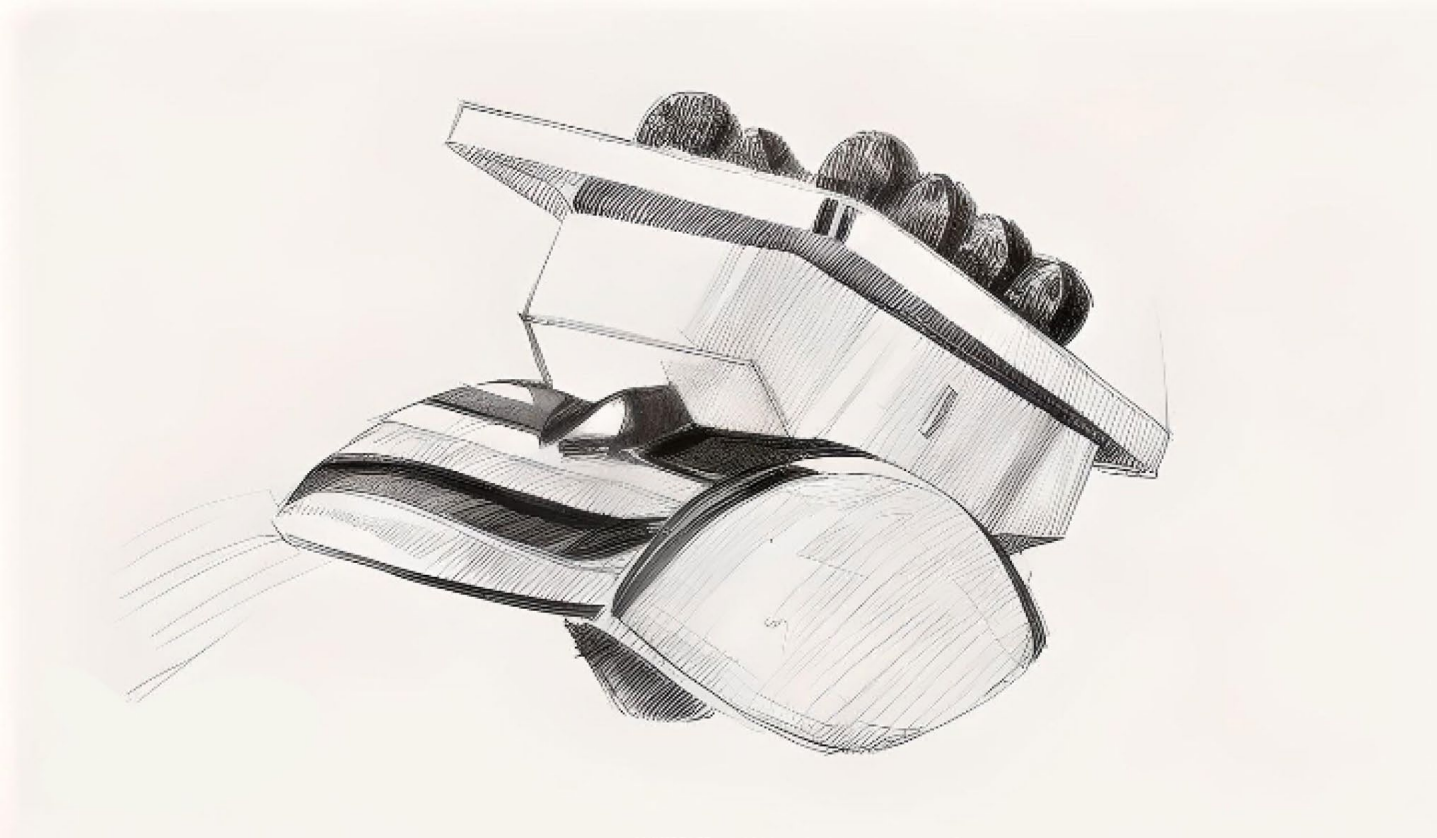
STAR (Scandinavian Total Ankle Replacement) prosthesis: a second-generation mobile-bearing design with semi-constrained articulation

The STAR prosthesis (Fig. 3) [54], developed by Håkon Kofoed in Denmark and first implanted in 1981, employed a MB that articulates superiorly with the tibial component, permitting unrestricted motion in all directions within the confines of the malleoli. Anteroposterior translation is managed at the tibial–meniscal interface, while rotation occurs between the insert and the talar component. However, this design precludes mediolateral rocking, and the dual articulation may predispose the insert to wear and dislocation. Malleolar impingement further increases the risk of fracture [57].

Subsequent iterations of the STAR were introduced in 1986 (cemented three-component configuration) and in 1990 (cementless three-component model) [54]. Anderson et al. reported a 70% survivorship at 5 years in a cohort of 51 implants, noting limitations in achieving sufficient dorsiflexion and recommending Achilles tendon lengthening when necessary [58]. Wood and Deakin [59], in a larger cohort of 200 patients, demonstrated a 92.7% survival rate at 5 years, with delayed wound healing and malleolar fractures as the most common complications. They also advocated for dorsiflexion of at least 10°, facilitated through Achilles tendon lengthening, and highlighted malalignment exceeding 15° in varus or valgus as a contraindication to TAR—a principle that later informed indications for secondary corrective procedures [60].

Introduced in the United States in 1981, the Buechel-Pappas prosthesis featured an uncemented MB design. Later modifications included a deeper talar sulcus and an additional fixation fin to mitigate talar subsidence [61]. In a 12-year follow-up of 40 patients treated with the original shallow sulcus design, 70% achieved good to excellent results, with a 20-year survivorship of 74.2%. In a separate series of 75 ankles with the deep sulcus modification, 88% demonstrated good to excellent outcomes and 92% survivorship at 12 years [62]. Doets et al., in a prospective observational study of 93 Buechel-Pappas implants with a mean follow-up of 8 years, reported an overall survivorship of 84%. The LCS (Low Contact Stress; originally developed by DePuy Synthes, Warsaw, IN, USA) Total Ankle System prosthesis—used earlier in 19 patients—showed only 60% survivorship at 10 years, whereas the subsequent Buechel-Pappas design reached 90% at 12 years [63].

Krishnapillai et al. [55] reported a 10-year survivorship of 86% (95% CI: 78–93%) for this system. Thirty-one patients (36%) underwent 55 reoperations, and 13 (15%) required revision. No statistically significant difference in 10-year survivorship was found between patients with inflammatory versus non-inflammatory joint disease (*p* = 0.47), nor between those with neutral versus non-neutral tibiotalar alignment (*p* = 0.16) [55].

The Agility Total Ankle System, introduced in 1984, featured a FB semi-constrained design and required syndesmotic arthrodesis to ensure load transfer through the fibula. Pyevich et al. evaluated the intermediate-term outcomes in the first 100 Agility ankle replacements performed by the original designer, F.G. Alvine. At a mean follow-up of 4.8 years (range: 2.8–12.3 years), 85 implants remained in 82 patients. Among these, 47 (55%) were pain-free and 24 (28%) reported only mild pain. The average ROM in the 56 patients examined was 36° (range: 10–64°), with 93% of cases deemed satisfactory [64].

In 2021, Bedard et al. published long-term results (minimum 20-year follow-up) of 132 Agility prostheses implanted between 1984 and 1994. Of the 126 available for follow-up, 17 (13.5%) underwent revision (n = 10) or arthrodesis (n = 7) for loosening. One additional revision was performed due to infection and another due to malposition of the talar component, resulting in a total revision rate of 15.1% [65].

### Third-generation implants

**Fig. 4:**
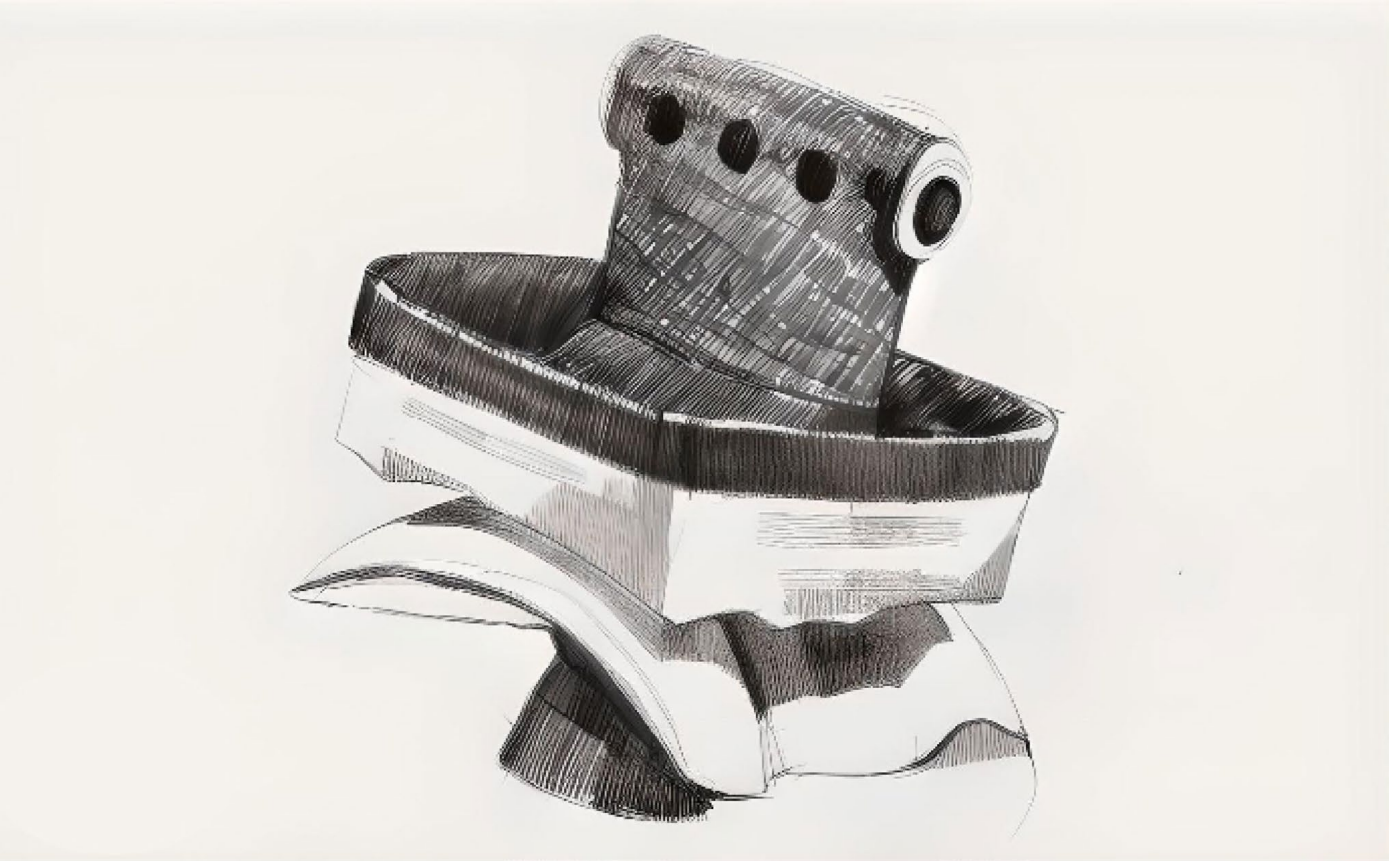
Salto™ prosthesis: a third-generation system with mobile bearings and minimal bone resection

Between the 1980s and 1990s, building on previous experiences, third-generation prostheses were developed to more accurately replicate the native anatomy and kinematics of the ankle joint. All were three-component systems composed of cobalt-chromium alloys, frequently featuring titanium porous coatings for cementless fixation, and in some cases, hydroxyapatite coatings. These implants incorporated a polyethylene MB and included models such as the HINTEGRA (Newdeal, Lyon, France) [66], Ankle Evolution System (AES; Zimmer-Biomet Europe, Belgium) [67], BOX (Bologna–Oxford; Finsbury, Leatherhead, UK) [68], Salto™ (Tornier, Montbonnot-Saint-Martin, France) (Fig. 4) [69], and Mobility (Depuy Synthes, Leeds, UK) [70]. Most of these designs were cementless and required either preservation of the talar dome or minimal talar resection. The use of a mobile polyethylene insert became more prominent. Emphasis shifted toward achieving soft tissue balance, exploiting ligamentous stability, correcting deformities, and defining salvage strategies for failed implants [52].

In 2022, Van Haecke et al. [71] reported clinical and radiographic outcomes of 97 HINTEGRA ankle replacements after a follow-up exceeding 5 years. The revision-free survival rate was 76%, and the explantation-free survival was 92%, with 10 cases requiring debridement and 5 undergoing component removal. Approximately 75% of patients reported no or only mild pain (*p* < 0.001). Radiographic evaluation demonstrated good positioning of the tibial components, while 87% of talar components were well-centered; posterior tibial calcifications were observed in 54.6% of cases.

In the late 1990s, the BOX (Bologna–Oxford) prosthesis was developed as a collaborative project between the Rizzoli Orthopaedic Institute and the Oxford Orthopaedic Engineering Centre, following detailed studies on ankle kinematics and the tendon-ligament apparatus [72]. Unlike the STAR prosthesis, the MB’s tibial surface in the BOX design is curved in both anteroposterior and mediolateral planes, to enhance congruency with the polyethylene insert [68]. The implant accommodates mediolateral tilting, reducing the risk of insert dislocation, although the potential for malleolar impingement persists [73].

Bianchi et al. [68], in 2021, conducted a follow-up analysis on 52 patients (54 BOX prostheses) from an initial cohort of 80 patients (82 prostheses), with a minimum follow-up of 10 years. Implant failure occurred in 20 of the 54 prostheses (37%). The mean Visual Analogue Scale (VAS) score decreased from 8.5 ± 1.2 to 2.9 ± 2.2 (*p* < 0.01), and the average American Orthopaedic Foot and Ankle Society (AOFAS) score improved from 28.6 ± 11.8 preoperatively to 72.7 ± 16.9 at the final follow-up (*p* < 0.01). Patient satisfaction was high, with 97% expressing satisfaction. The implant survival rate at the end of the follow-up period was 66%.

In 2020, Shih et al. [74] conducted a comparative analysis of TAR versus ankle arthrodesis using third-generation prostheses, drawing from three electronic databases. No statistically significant differences were observed in total AOFAS, pain, or alignment scores. However, TAR patients demonstrated significantly better functional AOFAS scores. VAS and AOS (Ankle Osteoarthritis Scale) total scores were comparable between groups. Gait analysis revealed no significant differences. The TAR group exhibited significantly better ROM and greater improvements, although it also showed higher complication and reoperation rates. Satisfaction levels were not significantly different between groups.

Recent systematic reviews have further clarified complication profiles and functional outcomes associated with third-generation implants. Vale et al. [75], analyzing 4,412 implants from 22 studies with a mean follow-up of 66.6 ± 40.9 months, reported a mean complication rate of 23.7% (range: 2.4–52%), with high-grade complications accounting for 35.6%. A statistically significant correlation was observed between complication severity and revision risk.

Hermus et al. [76], in a systematic review and meta-analysis comprising 127 studies and 16,964 implants (mean follow-up: 47.99 ± 29.18 months), identified intraoperative fracture (pooled incidence: 0.06; 95% CI: 0.04–0.08; GRADE: very low) and impingement (0.06; 95% CI: 0.04–0.08; GRADE: low) as the most common complications.

### Fourth-generation implants

**Fig. 5:**
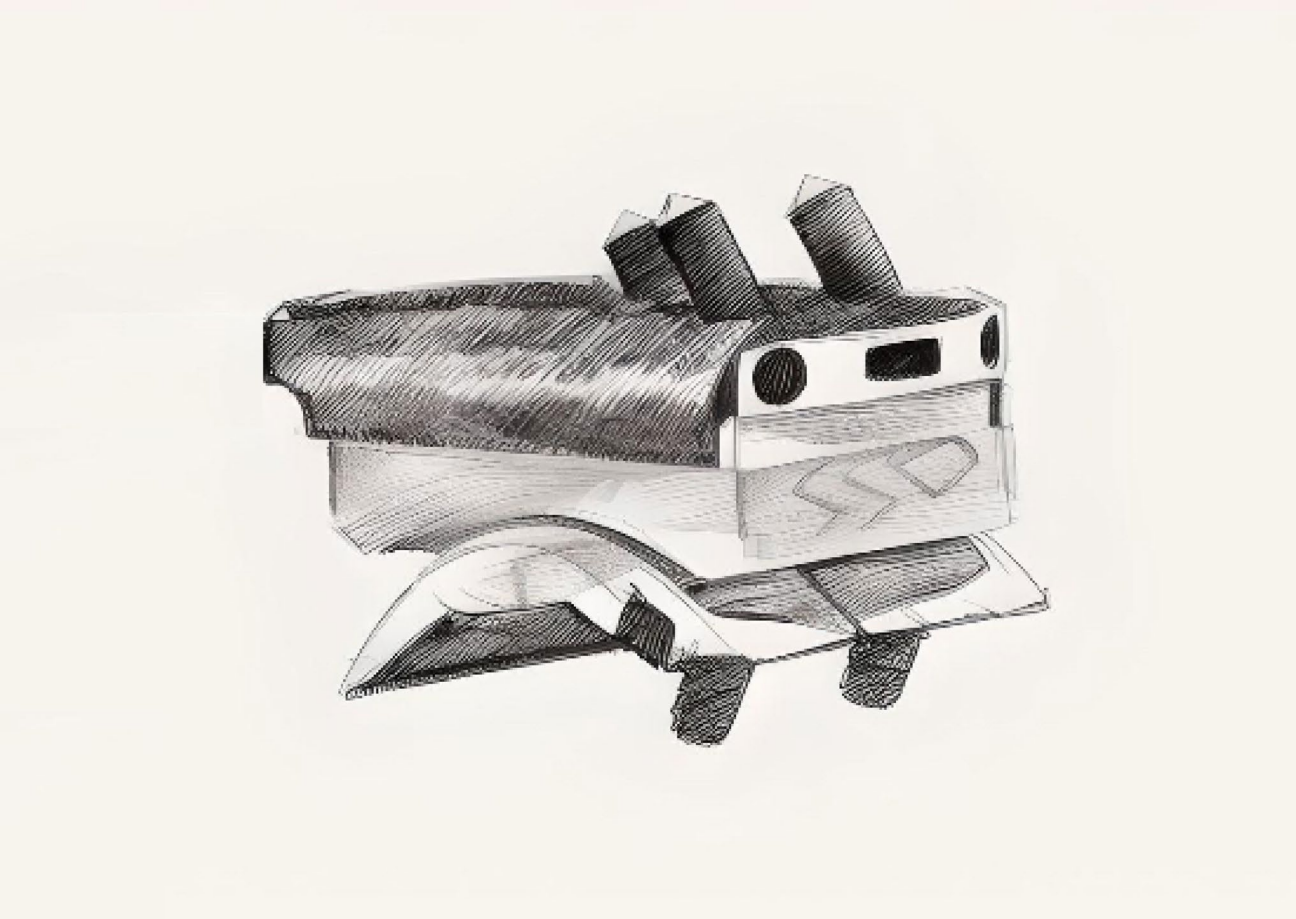
INFINITY prosthesis: a fourth-generation design optimized for minimal bone loss and improved fixation

The fourth generation of TAR systems represents the continued evolution of previous designs, with the primary aim of improving mechanical alignment, osseointegration, and surgical outcomes. In the United States, current fourth-generation models include the INFINITY (Stryker, Kalamazoo, MI, USA) (Fig. 5), CADENCE (Integra LifeSciences, Princeton, NJ, USA), VANTAGE (Exactech, Gainesville, FL, USA), Axiom (Kinos Medical, Wayne, PA, USA), Apex (Paragon 28, Englewood, CO, USA), and QUANTUM (In2Bones, Memphis, TN, USA) [77]. The Trabecular Metal (TM) Ankle System (Zimmer Biomet, Warsaw, IN, USA) also belongs to this generation and is unique in employing a lateral transfibular approach with a fibular osteotomy and a porous tantalum tibial component to enhance osseointegration [78].

These prostheses are characterized by streamlined tibial and talar components designed to minimize bone resection while maximizing bone–implant contact [79]. Although these systems are relatively recent, early clinical evidence indicates promising survivorship rates—ranging from 92 to 98%—along with marked improvements in pain and function during the first two postoperative years [77, 80, 81]. A comparative summary of reported survivorship rates by implant generation is provided in Table 1.

**Table 1:** Comparative survivorship rates of total ankle replacement implants

Generation	Implant name	Design type	Fixation	Follow-up (years)	Reported survivorship	Authors
1st	ICLH [40]	Constrained	Cemented	5.5	21% satisfactory cases	Bolton-Maggs et al. 1985
2nd	STAR [58]	Semi-constrained	Cementless	5	70%	Anderson et al. 2003
	STAR [59]	Semi-constrained	Cementless	5	92.7%	Wood and Deakin 2003
	Buechel-Pappas [63]	Mobile-bearing	Cementless	12	92%	Doets et al. 2006
	Agility [65]	Fixed-bearing	Cemented	20	84.9%	Bedard et al. 2021
3rd	BOX [68]	Mobile-bearing	Cementless	10	66%	Bianchi et al. 2021
	HINTEGRA [71]	Mobile-bearing	Cementless	≥ 5	76% (revision-free)	Van Haecke et al. 2022
4th	INFINITY [77]	Fixed-bearing	Cementless	~ 2	92–98%	Kim et al. 2022
	QUANTUM [82]	Fixed-bearing	Cementless	1	100% tibial/91.7% talar	Christie et al. 2025

Doyle et al. evaluated the radiographic incidence of heterotopic ossification in a cohort of 71 patients treated with selected fourth-generation TAR systems—specifically INFINITY, CADENCE, and VANTAGE; with an average radiographic follow-up of 19.7 months, the overall HO incidence was 69.0%, with the INFINITY prosthesis showing the highest rate [82].

**Fig. 6:**
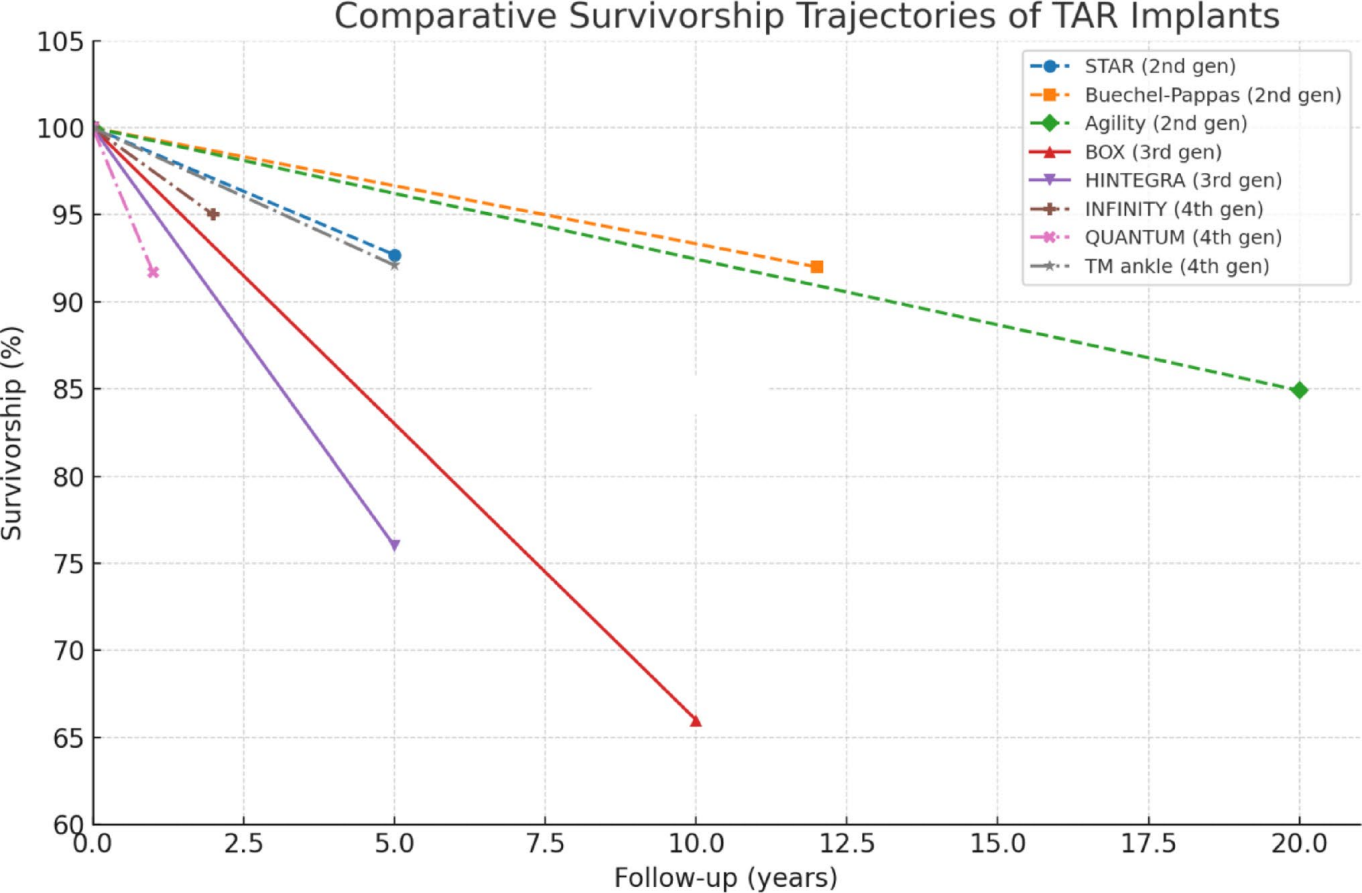
Comparative survivorship of total ankle replacement over time

In a 2025 study, Christie et al. reported early clinical outcomes of the Quantum implant over a minimum follow-up of 1 year. The implant demonstrated 100% survivorship for the tibial component and 91.67% for the talar component. Coronal plane deformities were corrected to neutral alignment in all cases. These findings suggest favorable early implant survivorship and satisfactory clinical and radiographic outcomes; however, longer-term follow-up is essential to determine the durability and performance of the device over time [83]. In contrast to these early series, Kim et al. [78] reported mid-term outcomes of the TM Ankle System in 38 cases, with a minimum 5-year follow-up. Survivorship was 92.1%, with a 7.9% revision rate and a 23.7% overall reoperation rate—predominantly for fibular hardware removal or medial gutter debridement—while all Foot and Ankle Outcome Score (FAOS) domains showed significant improvement. Figure 6 illustrates the comparative survivorship of selected TAR implants over time, based on clinical follow-up data.

Simultaneously, the field has advanced toward the development of TAR implants specifically designed for revision procedures. Historically, management options for failed TAR were limited to arthrodesis or below-the-knee amputation [61, 64, 84]. Currently, the INBONE II (Wright Medical, Memphis, TN, USA) system is increasingly used for revisions, although available options remain limited. As of now, INVISION (Stryker) and Salto Talaris XT (Integra LifeSciences) represent the only dedicated revision TAR systems, specifically engineered to address substantial bone loss and joint instability [85]. Nevertheless, clinical data on the performance of these revision-specific systems remain scarce, underscoring the need for further research. Given the growing interest and technological investment in this area, additional revision-specific solutions are likely to emerge in the near future.

Table 2 provides a generation-specific overview of the most commonly reported complications and failure mechanisms, including those associated with revision procedures.

**Table 2:** Common failure causes and complications by implant generation

Generation	Implant name	Main failure causes/complications	Authors
1st	ICLH [40]	Talar collapse, excessive bone resection, cement loosening	Bolton-Maggs et al. 1985
	Lord [34]	Complete talar resection, high mechanical failure	Lord and Marotte 1973
	Smith [41]	Ligament overload, malleolar fracture, talar collapse	Kirkup 1985
2nd	STAR [59]	Malleolar fracture, insert dislocation, edge loading	Wood and Deakin 2003
	Buechel-Pappas [55]	Talar subsidence, meniscal dislocation	Krishnapillai et al. 2019
	Agility [65]	Loosening, malalignment, infection	Bedard et al. 2021
3rd	BOX [68]	Malleolar impingement, implant failure	Bianchi et al. 2021
	HINTEGRA [71]	Posterior tibial calcification, talar component malposition	Van Haecke et al. 2022
4th	INFINITY, CADANCE, VANTAGE [81]	Heterotopic ossification (up to 69%), short-term data only	Doyle et al. 2023
	QUANTUM [82]	Early talar loosening (8.3%), unknown long-term complication rates	Christie et al. 2025

## Analytical correlation of design features and clinical outcomes

The historical progression of TAR systems, as chronicled across the four generations, provides a robust analytical dataset illustrating the critical correlation between specific design characteristics and measurable clinical performance metrics [81, 82]. A cross-generational analysis of the results synthesized in Table 1 (Survivorship) and Table 2 (Failure causes) allows for an objective assessment of design optimization over time and is essential for guiding contemporary surgical selection.

### The data-driven shift to cementless fixation

The data objectively support the widespread abandonment of cemented fixation. First-generation systems, characterized by constrained designs and reliance on cement (e.g., ICLH), demonstrated high failure rates, with cement loosening and subsequent talar collapse being the primary failure mechanisms [40]. The immediate and consistent transition to cementless fixation in second and subsequent generations was a direct, evidence-based response to mitigate the high incidence of these cement-related complications (Table 2) [38]. Current clinical practice, therefore, confirms cementless fixation as a fundamental and non-negotiable requirement for reliable outcomes in primary TAR [84].

### Critical comparison: fixed- versus mobile-bearing performance

The choice of bearing type represents the most significant design distinction in modern TAR and warrants critical analytical comparison. While the theoretical advantage of MB systems (e.g., STAR, HINTEGRA) lies in reducing shear forces and polyethylene wear by offering dual articulation, the historical and analytical data reveal a trade-off in failure modes. MB systems are often associated with unique complications, particularly insert dislocation and malleolar fracture (Table 2) [59], which directly impact long-term survivorship (e.g., BOX survivorship at 66% at 10 years) [68].

In contrast, the fourth-generation FB implants (e.g., INFINITY, QUANTUM), which represent the current evolution of TAR design, consistently report superior short- to mid-term survivorship rates (92% to 98%) (Table 1) [77, 83]. By eliminating the mobile polyethylene component, these designs effectively mitigate the specific mechanical failures associated with bearing dislocation [84].

### Objective guidance for contemporary implant selection

The synthesis of this analytical data provides direct guidance to the contemporary ankle surgeon, addressing the primary aim of this review. The highest documented reliability and most favorable failure profiles are consistently associated with fourth-generation, FB, cementless prostheses. These systems are designed to minimize bone resection while maximizing stability and predictable osseointegration. Therefore, the objective evidence from five decades of development strongly supports the selection of FB, cementless systems as the standard of care for the management of primary end-stage ankle OA.

## Limits of the study

This narrative review is subject to inherent methodological limitations. First, the narrative nature of the methodology lacks the structured rigor of systematic reviews or meta-analyses, introducing potential selection bias in the inclusion and interpretation of sources. The heterogeneity of the available literature—spanning different time periods, implant designs, and surgical techniques—further complicates direct comparisons and comprehensive data synthesis. This heterogeneity, particularly regarding differing follow-up durations and patient selection across studies, imposes inherent limitations on the direct analytical comparison of survivorship and failure rates between generations. Additionally, the reliance on historical reports and retrospective analyses restricts the generalizability of findings to current clinical practice. While the review provides an analytical comparison of published quantitative outcomes, the strength of the recommendations remains limited by the reliance on short- to mid-term data for the most recent fourth-generation implants. Furthermore, recent technological advances in TAR may not be fully represented due to delays in publication. Finally, although the assessment is based on expert interpretation, the potential for subjective bias in evaluating historical trends and technological evolution cannot be excluded.

## Conclusions

The historical progression of TAR provides a robust analytical framework for contemporary decision-making. Specifically, the data objectively demonstrate that the design shifts—from constrained, cemented implants (high failure due to loosening) to semi-constrained, MB designs (risk of insert dislocation) and finally to fourth-generation FB, cementless systems—directly correlate with improved survivorship (up to 98% at short-term follow-up). However, complications such as heterotopic ossification, talar loosening, and the technical demands of revision surgery continue to impact long-term success. These persistent challenges underscore the need for meticulous patient and implant selection based on documented performance metrics.

The availability of dedicated revision prostheses represents a promising development, though robust outcome data remain limited. Therefore, based on the synthesis of analytical performance data across five decades, this review provides objective guidance: fourth-generation, cementless prostheses are the system of choice for primary TAR due to their superior early reliability. Future priorities must focus on long-term data acquisition for these high-performing designs and on refining patient selection criteria to maximize functional restoration and long-term durability.

## Data Availability

No datasets were generated or analysed during the current study.
